# Research on Parameter Estimation Methods for Alpha Stable Noise in a Laser Gyroscope’s Random Error

**DOI:** 10.3390/s150818550

**Published:** 2015-07-29

**Authors:** Xueyun Wang, Kui Li, Pengyu Gao, Suxia Meng

**Affiliations:** 1 School of Instrumentation and Opto-electronics Engineering, Beihang University, No.37, Xueyuan Road, Haidian District, Beijing 100191, China; E-Mails: wangxueyun.buaa@gmail.com (X.W.); gaodiao008@163.com (P.G.); 2 School of Electric and Information Engineering, Beihang University, No.37, Xueyuan Road, Haidian District, Beijing 100191, China; 3 School of Automation Science and Electrical Engineering, Beihang University, No.37, Xueyuan Road, Haidian District, Beijing 100191, China; E-Mail: succeedwade@sina.com

**Keywords:** laser gyroscope, alpha stable noise, parameter estimation, probability density

## Abstract

Alpha stable noise, determined by four parameters, has been found in the random error of a laser gyroscope. Accurate estimation of the four parameters is the key process for analyzing the properties of alpha stable noise. Three widely used estimation methods—quantile, empirical characteristic function (ECF) and logarithmic moment method—are analyzed in contrast with Monte Carlo simulation in this paper. The estimation accuracy and the application conditions of all methods, as well as the causes of poor estimation accuracy, are illustrated. Finally, the highest precision method, ECF, is applied to 27 groups of experimental data to estimate the parameters of alpha stable noise in a laser gyroscope’s random error. The cumulative probability density curve of the experimental data fitted by an alpha stable distribution is better than that by a Gaussian distribution, which verifies the existence of alpha stable noise in a laser gyroscope’s random error.

## 1. Introduction

Despite a strong theoretical background and wide applications, Gaussian noise is inappropriate for modeling noise with an impulsive nature, which could be shown in the form of significant data mutation. In real circumstances, there are noises with a strong impulsive nature which the Gaussian model fails to describe, such as atmospheric noise caused by lighting, picture noise, radar noise and so on. These noises possess a common feature, namely their impulsive nature, which means that both the frequency and the magnitude of their data mutation are higher than what Gaussian noise describes. Another mathematical model has to be applied to such noise for better modeling. Alpha stable noise is impulsive in nature and has a heavy tail. It follows the alpha stable distribution and satisfies the Generalized Central Limit Theorem [1,2]. Alpha stable distribution is widely used to analyze and model signals for many reasons. Firstly, there exist many non-Gaussian signals with an impulsive nature and heavy tail in real life, including underwater signals, atmospheric environment signals, telephone line noise and some mobile communication signals [3,4,5,6,7]; secondly, alpha stable distribution has stable properties; thirdly, the Central Limit Theorem is popularized in cases where the second moment does not exist; and finally, there have been many successful applications in various areas [8,9,10,11,12], such as economics, physics, astronomy, biology, electrical engineering and so on.

As laser ring gyroscopes measure the angular rate through two beams of coherent lasers in a closed ring (light path) which is filled with mixed gases (generally helium and neon), data mutation for a laser ring gyroscope is much more severe than the Gaussian model describes. Therefore, alpha stable noise has been studied and found in a laser gyroscope’s random error based on experimental data with an impulsive nature and heavy tail. The alpha stable noise is determined by four parameters, so the accurate estimation of the parameters is the key to analyzing the signal. In this paper, three widely used estimation methods are analyzed in contrast with Monte Carlo simulation, and then the method with highest precision is used to estimate the parameters of alpha stable noise in a laser gyroscope’s random error based on experiment data. Note that the term “laser gyroscope’s random error” here is defined as the measurement error of the laser gyroscope. The cumulative probability density of experiment data of the laser gyroscope’s random error is fitted by alpha stable distribution with the estimated parameters, and the results show yet more evidence of the existence of alpha stable noise in the laser gyroscope’s random error.

The paper is organized as follows. In Section 2, the basic theory of alpha stable noise is briefly introduced. In Section 3, the widely used estimation methods, namely quantiles, empirical characteristic function and the logarithmic moment method are introduced in detail. In Section 4, the three methods are analyzed in contrast with Monte Carlo simulation. The method with the highest precision is applied to the experiment data to estimate the parameters of the alpha stable noise in the laser gyroscope’s random error. The existence of alpha stable noise in the laser gyroscope’s random error is verified in Section 5, and the conclusion is provided in Section 6.

## 2. Experimental Section Alpha Stable Noise

Alpha stable noise, also called Lévy noise, was put forward by Lévy when he studied the Generalized Central Limit Theorem. Compared with Gaussian distribution, alpha stable distribution is better when applied to describe non-Gaussian signals with impulsive properties and a heavy tail, because alpha stable distribution fits these properties better than Gaussian distribution. There is no closed-form expression for the probability density of alpha stable noise, so it can only be described by its characteristic function, which can be expressed as follows:

(1) $$\varphi (t)=\exp(it\mu -\gamma^{\alpha }|t{|}^{\alpha }(1-i\beta \mathrm{sgn}(t)\omega (t,\alpha )))$$

(2) $$\omega (t,\alpha )=\left\{ \begin{array}{l} \tan(\pi \alpha /2),\alpha \neq 1\\ 2\ln|t|/\pi ,\alpha =1 \end{array}\right.$$

where α (0<α≤2) is the characteristic exponent and the most important parameter characterizing alpha stable noise. The smaller the value of α is, the more severe the heavy tail of the distribution is, as is its impulsive nature. The parameter β is the symmetric parameter (−1≤β≤1). In the case of β=0, the distribution is called a symmetric alpha stable (SαS) distribution. The parameters μ and γ are the location parameter (−∞<μ<∞) and the scale parameter (γ>0), respectively. Therefore, a random variable X yielding an alpha stable distribution can be denoted as $X\text{\textasciitilde{}}S_{\alpha }(\beta ,\gamma ,\mu )$.

When α=2, the alpha stable distribution becomes a Gaussian distribution with mean μ and variance $\sigma^{2}=2\gamma^{2}$. In the cases of α=1,β=0 and α=1/2,β=1, the alpha stable distribution becomes a Cauchy distribution and a Lévy distribution respectively. Since alpha stable noise is determined by α,β,γ,μ, accurate estimation of the four parameters is essential to the analysis of alpha stable noise.

## 3. Parameter Estimation Methods of Alpha Stable Noise

The quantiles method, empirical characteristic function method and logarithmic moment method are three widely used methods of parameter estimation for alpha stable noise in engineering. They are introduced in detail and analyzed in contrast with Monte Carlo simulation in this section.

### 3.1. Quantiles Method

The quantiles method, which was put forward by Famma and Roll [13] and popularized by McCulloh in 1986 [14], can be used to estimate the characteristic exponent and scale parameter of a symmetric alpha stable distribution. The quantiles method could be applied to estimate the four parameters of alpha stable noise.

The characteristic exponent α and symmetric parameter β are consistently estimated according to Equation (3):

(3) $$\left\{ \begin{array}{l} \hat{\alpha }=\psi_{1}({\hat{v}}_{\alpha },{\hat{v}}_{\beta }),\hat{\beta }=\psi_{2}({\hat{v}}_{\alpha },{\hat{v}}_{\beta })\\ {\hat{v}}_{\alpha }=\frac{{\hat{x}}_{0.95}-{\hat{x}}_{0.05}}{{\hat{x}}_{0.75}-{\hat{x}}_{0.25}},{\hat{v}}_{\beta }=\frac{{\hat{x}}_{0.95}+{\hat{x}}_{0.05}-2{\hat{x}}_{0.5}}{{\hat{x}}_{0.95}-{\hat{x}}_{0.05}} \end{array}\right.$$

where ${\hat{x}}_{f}(f=0.05,0.25,0.5,0.75,0.95)$ is the sample data with which the quantile is calculated. The value of $\hat{\alpha }$ and $\hat{\beta }$ can be obtained by linear interpolation, denoted as $\psi_{1}$ and $\psi_{2}$, according to Equation (3) and Table II summarized by McCullohin [14].

With estimated $\hat{\alpha }$ and $\hat{\beta }$, scale parameter γ can be estimated as follows:

(4) $$\hat{\gamma }=\frac{{\hat{x}}_{0.75}-{\hat{x}}_{0.25}}{\phi_{3}(\hat{\alpha },\hat{\beta })}$$

where $\phi_{3}(\hat{\alpha },\hat{\beta })$ is the two-value function of $\hat{\alpha }$ and $\hat{\beta }$, which can be found in Table V detailed in reference [14]. With the linear interpolation method, the consistent estimation of γ can be obtained.

In order to estimate the location parameter μ, a new parameter ξ is introduced:

(5) $$\xi =\left\{ \begin{array}{l} \mu +\beta \gamma \tan\frac{\pi \alpha }{2},\alpha \neq 1\\ \mu ,\alpha =1 \end{array}\right.$$

The estimation of ξ is achieved by Equation (6):

(6) $$\hat{\xi }={\hat{x}}_{0.5}+\hat{\gamma }\phi_{5}(\hat{\alpha },\hat{\beta })$$

where $\phi_{5}(\hat{\alpha },\hat{\beta })$ is the two-value function of $\hat{\alpha }$ and $\hat{\beta }$, which can be obtained by linear interpolation according to Table VII in reference [14]. Then the location parameter μ can be estimated according to Equation (7) based on calculated $\hat{\xi }$.

(7) $$\hat{\mu }=\hat{\xi }-\hat{\beta }\hat{\gamma }\tan\frac{\pi \hat{\alpha }}{2}$$

### 3.2. Empirical Characteristic Function Method

Based on the law of large numbers, the sample characteristic function $\hat{\varphi }(t)$, which is the consistent estimation of Equation (1), can be calculated as follows:

(8) $$\hat{\varphi }(t)=\frac{1}{n}\sum_{k=1}^{n}\exp(jtx_{k})$$

where $\left\{x_{k}\right\}(k=1,2,\cdots n)$ is the sample of a random variable with independent and identical distribution and *n* is the number of samples. The equivalent expression of Equation (1) is as follows:

(9) $$\varphi (t)=e^{-|\gamma t{|}^{\alpha }}[\cos(\mu t-|\gamma t{|}^{\alpha }\beta sign(t)\omega (t,\alpha ))+j\sin(\mu t-|\gamma t{|}^{\alpha }\beta sign(t)\omega (t,\alpha ))]$$

Equation (10) can be easily achieved based on Equation (9):

(10) $$\log(-\log|\varphi (t){|}^{2})=\log(2\gamma^{\alpha })+\alpha \log(t)$$

According to the real and imaginary parts of φ(t) shown by Equation (9) in the condition of α≠1, Equation (11) can be obtained:

(11) arctan(Im(φ(t))Re(φ(t)))=μt−βγαtan(πα2)sgn(t)|t|α

The characteristic exponent α and the scale parameter γ can be obtained by linear regression estimation based on Equation (12):

(12) $$y_{k}=m+\alpha w_{k}+\varepsilon_{k},\begin{array}{c} \;k=1,2,\cdots ,K \end{array}$$

where $y_{k}=\log(-\log{\left(\hat{\varphi }(t_{k})\right)}^{2})$, $m=\log(2\gamma^{\alpha })$, $w_{k}=\log(t_{k})$, $\varepsilon_{k}$ is the random error, and k of the real data sets $t_{k}=\pi k/25$ (k=1,2,⋯,K) can be accessed according to the Table I in reference [15].

**Figure 1:**
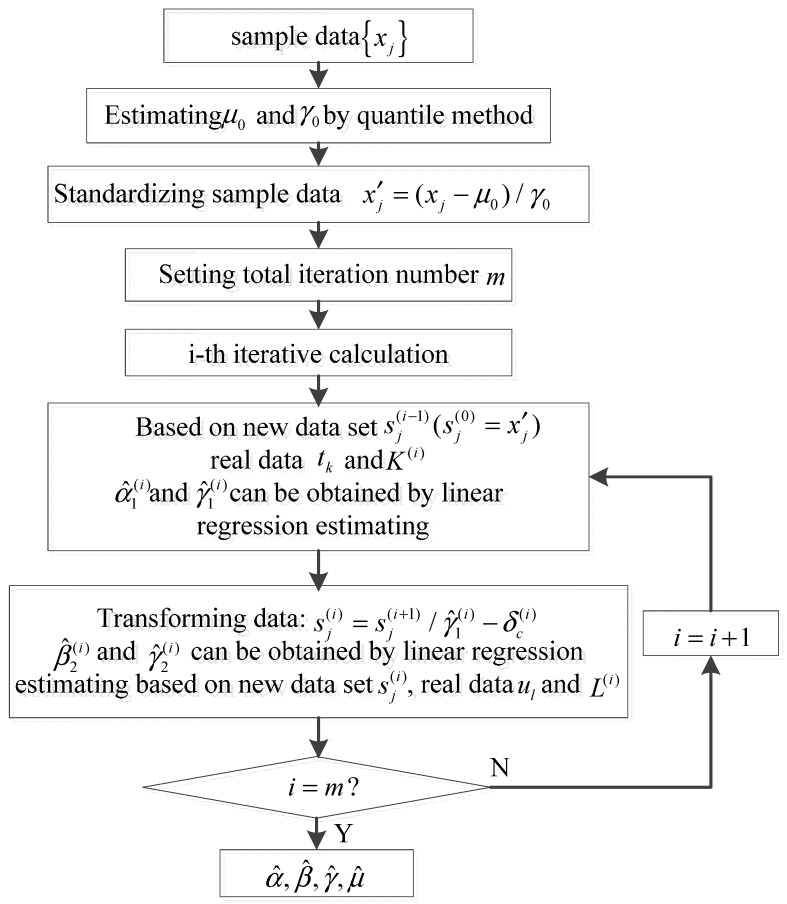
Calculation process of ECF method.

With the estimated α and γ, the symmetric parameter β and location parameter u can be obtained by linear regression estimation based on Equation (13):

(13) $$z_{l}=\mu u_{l}-\beta \gamma^{\alpha }\tan(\pi \alpha /2)sign(u_{l})|u_{l}{|}^{\alpha }+\eta_{l},\begin{array}{c} \;l=1,2,\cdots L \end{array}$$

where $z_{l}=g_{n}(u_{l})+\pi k_{n}(u_{l})$, gn(u)=arctan(Im(φ(u))/Re(φ(u))), $\eta_{k}$ is the random error, and L of the real data sets $u_{l}=\pi l/50$ (l=1,2,⋯,L) can be searched in Table II in reference [16]. The detailed process of ECF method is shown in Figure 1.

### 3.3. Logarithmic Moment Method

The statistical moments of alpha stable distribution are finite only in the case of p<α. The fractional lower order moment method and logarithmic moment method were put forward in [17] and they are applicable for symmetric alpha stable distribution. The methods were then expanded by Kuruolu [18] to be suitable for any alpha stable distribution. The advantage of the logarithmic moment method over the fractional lower order moment method is that the logarithmic moment method avoids the difficulty in solving function sin*c* and selecting the value of *p*.

The introduction of the negative order moments makes it possible to calculate any order derivative of $E[|X{|}^{p}]$ which is continuous at *p* = 0, and the moment generation function is defined as follows:

(14) $$E[{\left|X\right|}^{n}]=\underset{p\to 0}{\lim}\frac{d^{n}}{dp^{n}}(E[{\left|X\right|}^{p}]),\begin{array}{cc} & n=1,2,3\cdots \end{array}$$

Assume $X\sim S_{\alpha }(\beta ,\gamma ,0)$, then:

(15) $$L_{1}=E[\log|X|]=\varphi_{0}(1-\frac{1}{\alpha })+\frac{1}{\alpha }\log|\frac{\gamma }{\cos\theta }|$$

(16) $$L_{2}=E[{\left(\log|X|-E[\log|X|]\right)}^{2}]=\varphi_{1}(\frac{1}{2}+\frac{1}{\alpha^{2}})-\frac{\theta^{2}}{\alpha^{2}}$$

(17) $$L_{3}=E[{\left(\log|X|-E[\log|X|]\right)}^{3}]=\varphi_{2}(1-\frac{1}{\alpha^{3}})$$

where $\varphi_{0}=-0.57721566$, $\varphi_{1}=\pi^{2}/6$, $\varphi_{2}=1.2020569$, and

(18) θ=arctan(βtan(απ/2))

$L_{2}$ is often used to obtain the parameter α by applying centro-symmetrization [18] to the obverted data, since the moments of higher order tend to be more noisy. Therefore α can be estimated by:

(19) $$\alpha ={\left(\frac{L_{2}}{\varphi_{1}}-\frac{1}{2}\right)}^{-1/2}$$

Based on the estimated α and the obverted data by centering, Equation (20) can be derived:

(20) $$|\theta |={\left((\frac{\varphi_{2}}{2}-L_{2})\alpha^{2}+\varphi_{1}\right)}^{1/2}$$

Then, $\left|\beta_{0}\right|$ can be estimated according to Equation (18). Note that if centering is applied, it is necessary to transform $\left|\beta_{0}\right|$ by multiplying $\left(2+2^{\alpha })/(2-2^{\alpha }\right)$ to obtain |β| and the sign of β is determined by Equation (21):

(21) $$\begin{array}{l} K=sign(|X_{\max}-X_{md}|-|X_{\min}-X_{md}|)\\ \beta =K|\beta | \end{array}$$

where $X_{\max},X_{md},X_{\min}$ are the maximum, median and minimum of original data respectively.

Estimate $L_{1}$ for the centered data and the scale parameter $\gamma_{0}$ is obtained by Equation (22) with estimated α and β:

(22) $$\gamma_{0}=|\cos(\theta )|\exp((L_{1}-\varphi_{0})\alpha +\varphi_{0})$$

To get the scale parameter γ of original data, $\gamma_{0}$ has to be transformed into $\gamma_{0}$ by dividing $\left(2-2^{1/\alpha }\right)$.

The local parameter μ is estimated according to Equation (23)

(23) $$\mu =\mu_{0}{\left(2-2^{1/\alpha }\right)}^{-1}$$

where $\mu_{0}$ is the median of the obverted data with β=0 by applying the unbiased process [2].

## 4. Method Comparison

To compare the estimation accuracy of the three methods above, they are applied to the data featured with an alpha stable distribution whose parameters are known. The data is generated by the CMS method introduced in reference [19]. The values of the four parameters that describe alpha stable noise are the means of 1000 estimations through Monte Carlo simulation.

**Figure 2:**
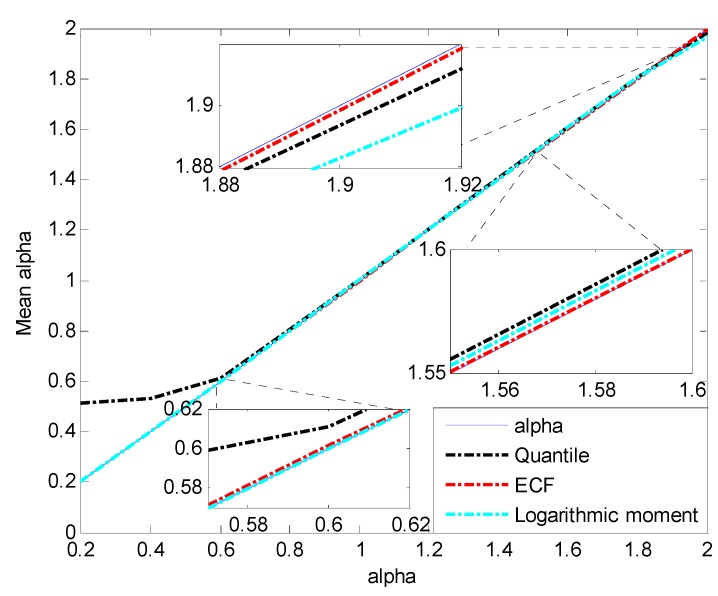
Mean of estimated α.

**Figure 3:**
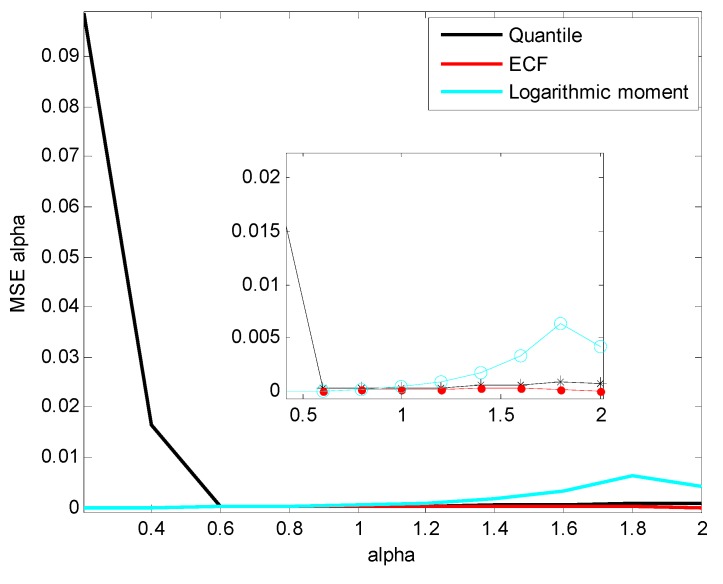
MSE of Estimated α.

Random variable $X\text{\textasciitilde{}}S_{\alpha }(0.5,1.5,0)$ is generated with the CMS method. The number of each simulation sample is 10,000, and the parameter α increases from 0.2 to 2 with the step of 0.2. The three methods mentioned in previous contents are applied to the simulation data to compare the estimation accuracy of α. The mean and mean square error (MSE) of estimated α are shown in Figure 2 and Figure 3.

Figure 2 and Figure 3 show that the value of α estimated by the quantile method diverges greatly (an obviously large MSE compared to other methods) in the case of α<0.6. From α=1.2, the estimation MSE of logarithmic moment increases and reaches the maximum at α=1.8, while the corresponding estimation MSEs of the quantile method and ECF method remain small and stable.

**Figure 4:**
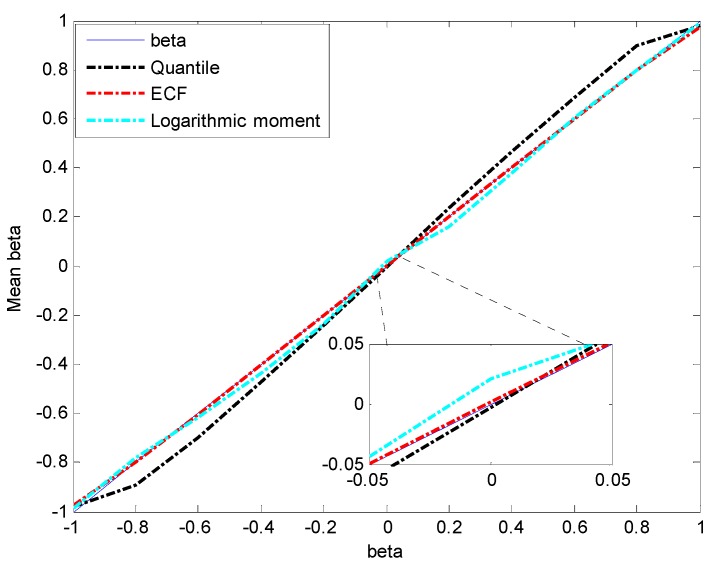
Estimated mean of β.

**Figure 5:**
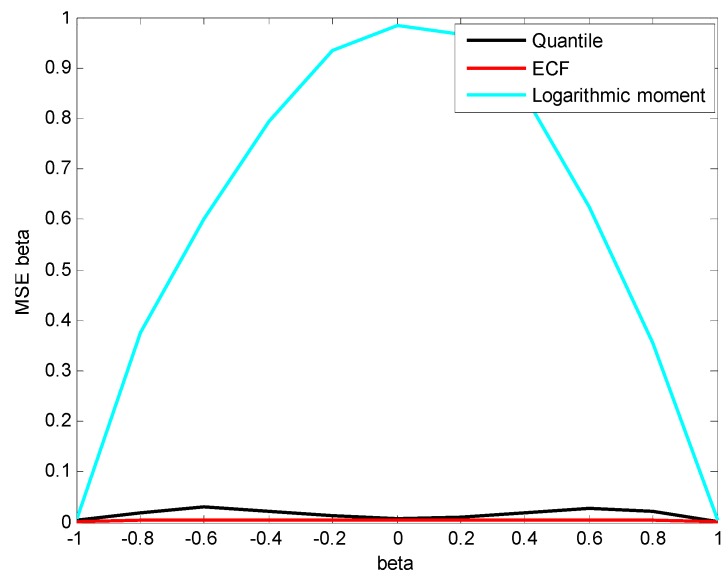
Estimated MSE of β.

In order to analyze the estimation accuracy of β, the three methods are applied to estimate the parameters of simulation data $X\text{\textasciitilde{}}S_{1.8}(\beta ,1.5,0)$ whose value of β increases from −1 to 1 with a step of 0.2. The results are depicted in Figure 4 and Figure 5.

From Figure 4 and Figure 5, it is clear that the ECF method has the highest estimation accuracy and the greatest stability for β. Although the mean of β estimated by the logarithmic moment method is close to the true value, the shape of estimation MSE curve is like a bell (Figure 5) and the maximum is reached at β=0, which clearly indicates the instability of the logarithmic moment method.

**Figure 6:**
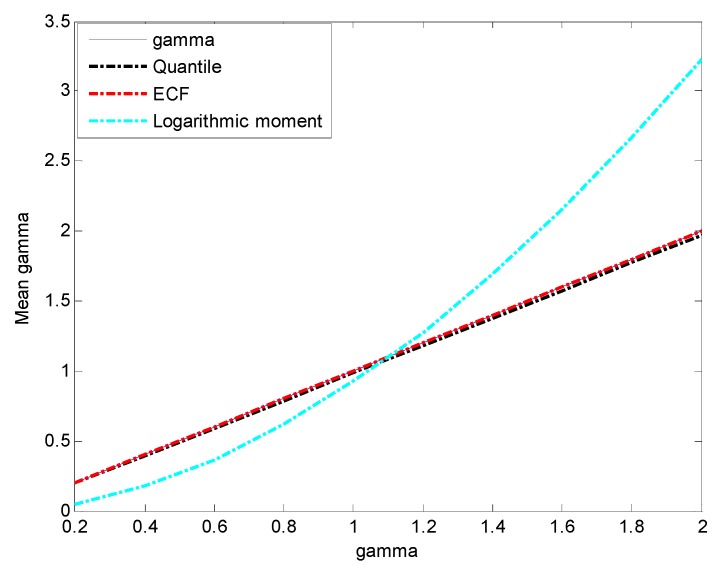
Estimated mean of γ.

**Figure 7:**
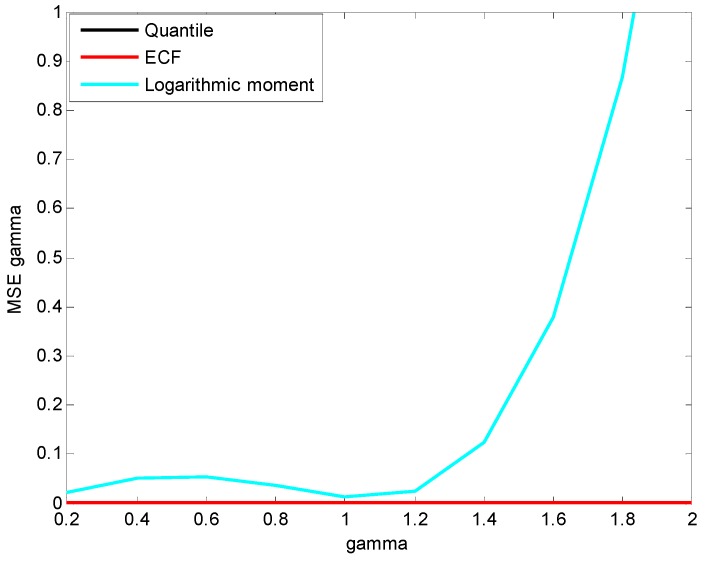
Estimated MSE of γ.

The random variables $X\text{\textasciitilde{}}S_{1.8}(0.5,\gamma ,0)$ and $X\text{\textasciitilde{}}S_{1.8}(0.5,1.5,\mu )$ are generated respectively for the comparisons of the estimation accuracy of scale parameter γ and location parameter μ. The means and MSEs of the estimations are shown in Figure 6 and Figure 7.

**Figure 8:**
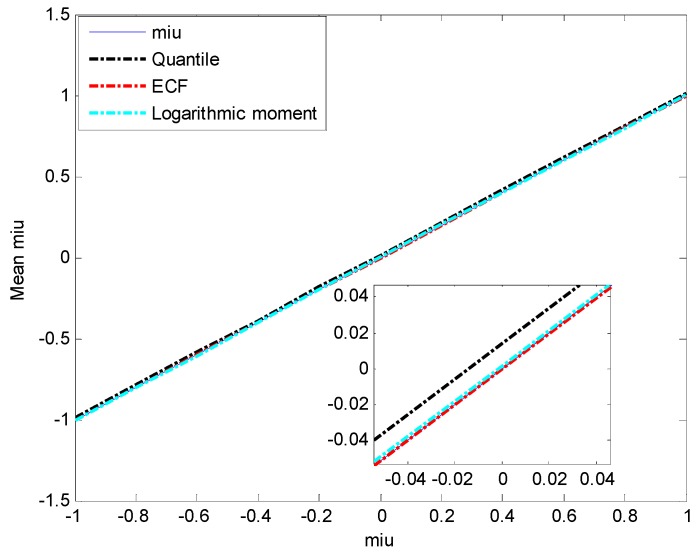
Estimated mean of μ.

**Figure 9:**
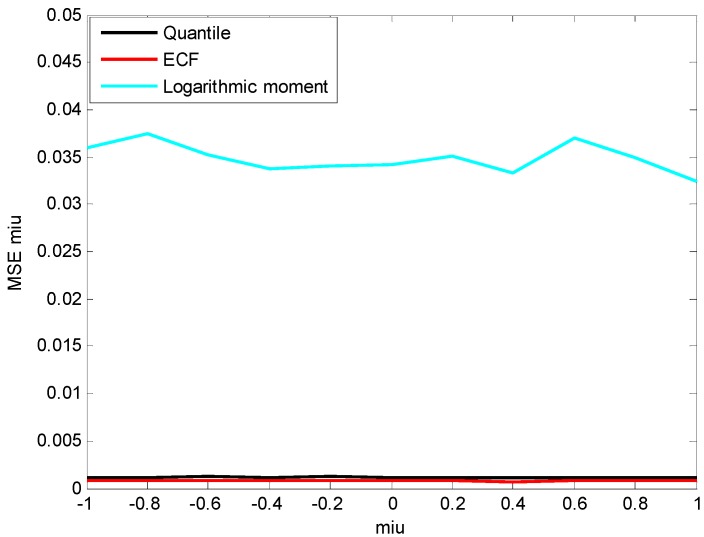
Estimated MSE of μ.

Figure 6 and Figure 7 show that the quantile method and ECF method have better estimation accuracy for γ than the logarithmic moment method, whose estimation mean diverges greatly, and the estimation MSE shows that the divergence starts from γ=1.2. As for the estimation accuracy of μ, it is easily found in Figure 8 that the means of μ estimated by the three methods are all very close to the true value, while the estimation MSEs of the quantile method and ECF method are much smaller than that of the logarithmic moment method, as shown in Figure 9.

To sum up, the quantile method, which has little calculation and high estimation accuracy for the scale parameter and location parameter, is suitable for estimating parameters of alpha stable noise whose characteristic exponent α satisfies 0.6<α≤1.2. However, the logarithmic moment method is just suitable for estimating parameters of alpha stable noise with α≤1.2, since the estimation MSE increases significantly when α>1.2. The reason is that, with centro-symmetrization being applied, the number of valid samples decreases to only half of the original number. The estimations of symmetric parameter β and scale parameter γ diverge from true values too, because the number of valid samples decreases to 1/2 and 1/3 of the original number, respectively. Moreover, the estimation error of α will degrade the accuracy of the other three parameters according to Equations (20)–(23).

By comparative analysis, the ECF method has the best estimation accuracy for all four parameters of alpha stable noise, along with best stability and without any limitations, followed successively by the quantile method and the logarithmic moment method.

## 5. Parameter Estimation of Alpha Stable Noise in a Laser Gyroscope’s Random Error

To verify the existence of alpha stable noise in a laser gyroscope’s random error in solid, it is necessary to analyze experimental data. We estimate the parameters of alpha stable noise in a laser gyroscope’s random error based on experimental data and then plot the cumulative probability density curve of the experiment data with the estimated parameters. Based on the comparisons above, the empirical characteristic function method (ECF) is applied to the 27 groups of experimental data for laser gyroscopes.

**Figure 10:**
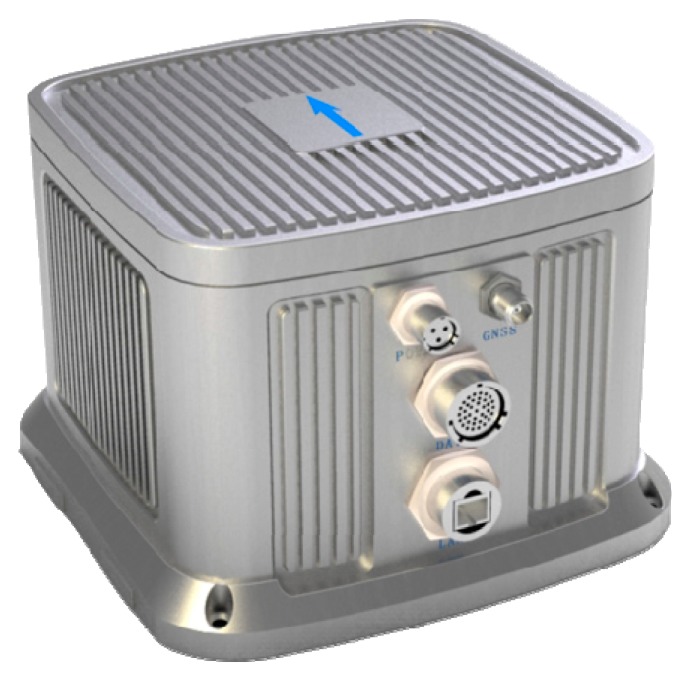
Inertial Navigation System G-380 by Sanetel.

The experimental data was obtained from G-380, an inertial navigation system (INS) based on laser gyroscopes and produced by Sanetel, as shown in Figure 10. The basic principle of this selected laser ring gyroscope is the Sagnac Effect. A laser source produces two beams of coherent lasers which transfer in opposite directions in a closed ring (light path). The closed ring is a triangle and filled with mixed gases. When the gyroscope is static, the time that the lasers take to traverse the ring in the two directions is the same. However, when the gyroscope rotates, the time that the lasers take to traverse the ring in the two directions is different according to the Sagnac Effect. This time difference introduces a tiny separation between the frequencies of the two opposite-transferring lasers and the angular rate can be measured based on the difference in the frequencies. The specifications of the INS and its gyroscopes provided by the manufacturer are shown in Table 1. In all 27 experiments, the INS was fixed and static on a platform in a laboratory at Beihang University, with the temperature ranging from 20° to 25° centigrade. The sample rate was 1000 Hz and each experiment lasted at least 1 h. The experimental data was used for parameter estimation of alpha stable noise to verify the existence of alpha stable noise in laser ring gyroscopes. Moreover, the complementary cumulative distribution function (CCDF), which could show the cumulative probability density of the experimental data, was calculated and plotted to compare with that of alpha stable noise and Gaussian noise.

**Table 1:** Specifications of gyroscope provided by the producer.

Specifications	Bias Repeatability	Scale Factor Nonlinearity	Temperature Range	Angular Input Range
	≤0.01 °/h	≤20 ppm	−40 °C~60 °C	±300 °/s

The parameter estimation results are listed in Table 2. It can be found that the value of alpha is between 1.88 and 1.98, with a mean of 1.95 (smaller than 2), which demonstrates that it is the alpha stable noise that exists in laser gyroscope’s random error, rather than the Gaussian noise. The standard deviations (STD) of all estimations show that the means of the estimated parameters are within a small range of error, especially the parameter alpha.

**Table 2:** Parameters estimated by ECF of alpha stable noise in 27 groups experiment data.

Group	Parameters			
	α	β	γ	μ
1	1.884387469927	0.29048748570638	1.19431932508 × 10^−6^	1.135547379819 × 10^−8^
2	1.940966655721	0.47092353950084	1.01585846371 × 10^−6^	8.906361125893 × 10^−9^
3	1.944815699729	−0.2938713913071	8.33981468432 × 10^−7^	−8.25321937116 × 10^−9^
4	1.9529396243278	0.177780856516045	8.215567199174 × 10^−7^	1.942705866842 × 10^−9^
5	1.9714966676089	0.432377047434275	9.139798869075 × 10^−7^	3.283604482693 × 10^−9^
6	1.9499370592781	0.277004383309236	8.897195807798 × 10^−7^	3.925822304770 × 10^−9^
7	1.9546056811054	0.606478176711532	9.634580646817 × 10^−7^	5.769601813242 × 10^−9^
8	1.9510495547351	0.561007146599921	9.103727987938 × 10^−7^	6.977343907612 × 10^−9^
9	1.9452900872635	0.389701598706903	9.086608483011 × 10^−7^	2.229129782177 × 10^−9^
10	1.9635266249299	0.442081734459963	9.562509780293 × 10^−7^	5.265916942076 × 10^−9^
11	1.9784044698261	0.147806605220355	8.662052745872 × 10^−7^	1.892428696282 × 10^−10^
12	1.9557050675943	−0.02482694833650	7.915732594649 × 10^−7^	−1.55428146059 × 10^−9^
13	1.9654304588139	−0.19449488439177	6.850711132306 × 10^−7^	−2.46589348188 × 10^−9^
14	1.9613917650785	0.308003095966491	7.459305467438 × 10^−7^	1.777108010607 × 10^−9^
15	1.9490905244952	0.182440223456915	7.217029380462 × 10^−7^	1.664885256641 × 10^−9^
16	1.9550153328475	−0.26087169286443	6.937094061097 × 10^−7^	−2.23207641529 × 10^−9^
17	1.9739102216051	−0.42225108427118	7.503156258410 × 10^−7^	−3.21815653702 × 10^−9^
18	1.9805246530289	−0.23490912096448	7.209679368495 × 10^−7^	−1.36661789630 × 10^−9^
19	1.9830793176829	0.239257324701739	6.968329274752 × 10^−7^	9.797373972322 × 10^−10^
20	1.9801436990134	0.001984626806417	7.507732635323 × 10^−7^	7.781496618892 × 10^−10^
21	1.9782996750290	−0.37116757112550	6.214628700519 × 10^−7^	−1.42265330938 × 10^−9^
22	1.9565833941126	−0.14156589731890	6.036147577533 × 10^−7^	−7.02904004332 × 10^−10^
23	1.9612460510221	−0.13214349790335	6.899181310174 × 10^−7^	−1.04021534031 × 10^−9^
24	1.9567985561225	0.059587947531770	6.955555472553 × 10^−7^	4.901300506776 × 10^−10^
25	1.9714506339453	−0.23286450800332	7.090481572823 × 10^−7^	−1.57505083247 × 10^−9^
26	1.9669221913582	−0.36253860301427	7.463910944024 × 10^−7^	−3.04459122018 × 10^−9^
27	1.961991642863	−0.16018117128825	7.452332315853 × 10^−7^	−2.76627094778 × 10^−9^
**M × 10an**	**1.9590741774525**	**0.0650087195467**	**8.01573634146 × 10^−7^**	**9.575432990901 × 10^−10^**
**STD**	**0.0191504675654**	**0.31261315756723**	**1.34542145660 × 10^−7^**	**4.172576421688 × 10^−9^**

On the basis of parameter estimation of alpha stable noise, the complementary cumulative distribution function (CCDF) was calculated. CCDF is the complement of the cumulative distribution function (CDF), namely CCDF = 1 − CDF. Therefore, CCDF could also describe the cumulative probability density of a random variable as CDF does. The CDF of a random variable *X* evaluated at point *a* is defined as the probability that *X* will take on values that is no larger than *a*. It is represented, in a plot, by the area under the probability distribution function (PDF) to the left of *a*. The CCDF of the experimental data was calculated, as were the CCDFs of Gaussian noise and alpha stable noise based on the estimated parameters previously mentioned. The three CCDFs are compared to further prove that alpha stable noise does exist in laser ring gyroscopes.

**Figure 11:**
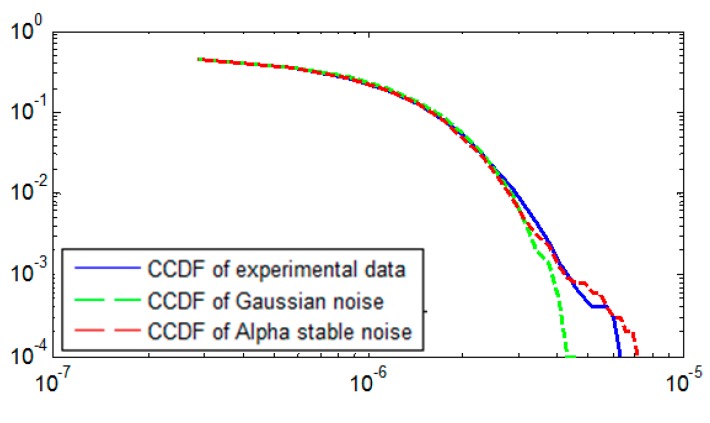
The CCDF fitting curve of the 12th group experiment data.

As all groups of data show a similar result and draw the same conclusion, but only the 12th experiment is detailed and plotted as an example. Figure 11 simultaneously depicts the CCDFs of the 12th group experiment data fitted by Gaussian distribution and alpha stable distribution. The real CCDF of experimental data is also shown as the reference. The CCDFs are plotted in log-log. The X axis represents the log of the random error log(*X*), while the Y axis represents the log of CCDF log(1 − *F*(*X*)). When log(*X*) > 2 × 10^−6^, the CCDF of Gaussian distribution separates with the real CCDF, ending at log(*X*) = 4 × 10^−6^. However, the CCDF of alpha stable distribution follows the real CCDF till log(*X*) = 7 × 10^−6^. For a random variable with heavy tail behavior, when the random variable is large, its CCDF is much greater than that of a random variable without heavy tail behavior, such as Gaussian distribution. Therefore, the result clearly demonstrates that alpha stable distribution fits the reality better, and it is more applicable to be used to describe the heavy tail behavior of a laser gyroscope’s random error than Gaussian distribution. This result further verifies the existence of alpha stable noise in a laser gyroscope’s random error.

Although a better noise model of the alpha stable noise model is established and proved for laser gyroscope’s random error in this paper, effective measures and methods to eliminate or reduce the alpha stable noise are not detailed here, because alpha stable noise is still a random noise which could be effectively suppressed by traditional low-pass filter technology. Such technology is not anything new, so we do not discuss in detail it in this paper.

## 6. Conclusions

The quantile method, empirical characteristic function method and logarithmic moment method are the most widely used methods to estimate parameters of alpha stable noise. They are comparatively analyzed through theoretical analysis and Monte Carlo simulation in this paper, and the results illustrate that the ECF has the highest estimation accuracy and stability, followed successively by quantile method and the logarithmic moment method. The parameters of alpha stable noise in real data of laser gyroscopes are estimated using the ECF method, and the mean of parameter alpha is 1.96, proving that alpha stable noise exists in a laser gyroscope’s random error. Furthermore, the complementary cumulative distribution function (CCDF) of the experiment data is calculated. The CCDF of alpha stable distribution based on the estimated parameters fits the real CCDF better than that of Gaussian distribution, especially for the heavy tail behavior. This result further verifies the existence of alpha stable noise in a laser gyroscope’s random error.
